# Successful treatment of Gustilo type 3C open tibial fracture with a massive muscles and soft tissues wasting: A case report

**DOI:** 10.1016/j.amsu.2022.103580

**Published:** 2022-04-04

**Authors:** Abdullah Alsultan, Mohamad Al Masri, Maher Al-Hajjaj, Muhammad Al Atrash, Mohammad Alsultan

**Affiliations:** a Department of Orthopedic Surgery, Al Assad and Al Mouwasat University Hospitals, Damascus University- Faculty of Medicine, Damascus, Syria; b Department of Urology, Aleppo University Hospital, Aleppo, Syria; c Department of Orthopedic Surgery, Damascus University- Faculty of Medicine, Khorfakkan Hospital, Sharjah, United Arab Emirates; d Department of Nephrology, Al Assad and Al Mouwasat University Hospitals, Damascus University- Faculty of Medicine, Damascus, Syria

**Keywords:** Gustilo type 3C, Open tibial fracture, Cross-leg flap, Case report

## Abstract

**Introduction and importance:**

Gustilo-Anderson type 3 open tibial fractures are commonly accompanied by extensive damage and serious complications, especially in types 3B and 3C.

**Case presentation:**

A 25-year-old male was transferred after motor vehicle accident and the emergent choice was an above-knee amputation in other two hospital. A left open distal tibial fracture (Gustilo type 3C) with a wide and contaminated soft tissues defect, that extended from the knee to the midfoot, is accompanied by wasting of the anterior compartment muscles and ruptured of the anterior tibial artery. We did irrigation and debridement and then we placed an anteriomedial external fixation system type AO with pins. A cross-leg flap and free skin grafts from the opposite limb was performed three weeks after a daily irrigation and debridement, povidon and ozone cream bandages, and antibiotics. After twelve months of follow-up, the fracture was healed and the external fixator was removed.

**Discussion/conclusion:**

Gustilo-Anderson type 3 open fractures remain a veritable orthopedic challenge, even for surgeons with greater experience, because of neurovascular damages, high amputation rate, and vast soft tissue injuries. The collaboration of the multidisciplinary surgical team led to preserve the limb with good result after one year. It is reasonable to judge and attempt a limb preservation even with wide open tibial fractures.

## Highlights

• Open tibial fracture (Gustilo type 3C) with an extended loss of muscles and soft tissue does not mean to amputate.
• Delayed cross-leg flap and free skin grafts can be performed for open contaminated wound fractures.
• Intraoperative and postoperative assessment of the injured artery by doppler will allow to preserve the limb circulation.

## 1 Introduction

The incidence of open tibial fracture, as a part of isolated injury or polytrauma, is on a rise due to increase in the incidence of motor vehicle accidents [1]. The prevalence of tibial shaft fractures is estimated by 67% [2]. Gustilo-Anderson type 3 open tibial fractures are commonly accompanied by serious complications such as amputation, infection, nonunion, malunion, and soft tissue losses [3,4]. Also, it was found that the anterior tibial artery is more commonly injured (31.9%) in comparison with the posterior tibial artery (8.9%) [5].

The extensive damage seen in types 3B and 3C may be a veritable challenge, even for surgeons with greater experience. Where types 3C (75%) and 3B (25%) predominated among patients subjected to secondary amputation. It requires a clinical decision between attempts to salvage the limb and amputation. Clinical advances in orthopedic, plastic and vascular surgery have provided more options for reconstructing injuries to limbs that, around 20 years ago, would have resulted primarily in amputation [2]. There are now studies suggesting that salvage is a cost-saving strategy, that offers a better quality of life than amputation [6].

Here, we report a challenging and successfully treated case of a left tibial fracture (Gustilo type 3C) with an extended loss of muscles and soft tissues of the lower limb. Moreover, the massive and contaminated wound was successfully treated with a daily irrigation and debridement that followed by a cross-leg flap and free skin grafts from the opposite limb after orthopedic surgery. This case report examines one such presentation in line with the SCARE guidelines [7].

## 2 Presentation of case

A 25-year-old male was transferred to our emergency department 6 h after motor vehicle accident. He had left lower limb crushing injury and the patient with his family refused above-knee amputation procedure in two other hospitals. He had a personal history of smoking.

**Fig. 1:**
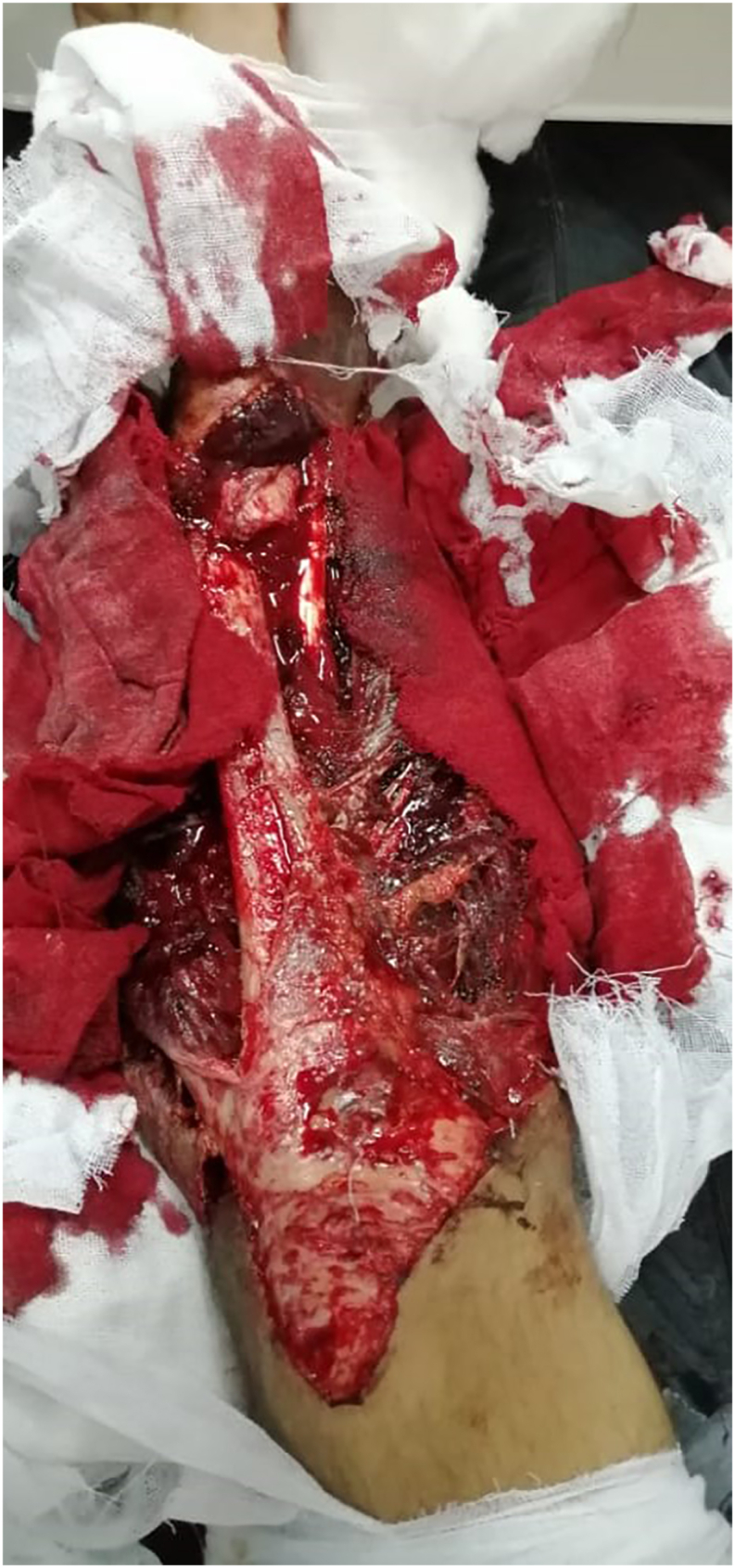
**Gustilo type 3C**; left open distal tibial fracture with a wide and contaminated soft tissues defect, and anterior compartment muscles wasting.

The physical examination revealed a left open distal tibial fracture (Gustilo type 3C) with a wide and contaminated soft tissues defect, that extended from the knee to the foot, and accompanied by wasting of the anterior compartment muscles, which consisted of tibialis anterior, extensor digitorum longus, extensor hallucis longus and peroneus tertius (Fig. 1). Also, ankle dorsiflexion was absent with an intact planter flexion and intact sensation in the left foot and posterior tissue of the left leg. The Orthopedic Team placed an external fixator to stabilize the fracture site.

No other concomitant or life-threatening injuries were detected and vital signs were as follow: blood pressure was 90\50 mmHg, pulse was 140 beats\ min, oxygen saturation was 96%, and Glasgow coma scale was 15\15. Laboratory tests in emergency were as follow: white blood cells 9400/ml, hemoglobin 9.1 g\ dl, platelet 426 th/m, sodium 136 mg\dl, potassium 3.7 mg\dl, urea 29 mg\dl, creatinine 0.9 mg\dl, and prothrombin time activity 94%.

Under epidural anesthesia, vascular surgeons presented during the procedure and a careful surgical exposure revealed that the anterior tibial artery was completely ruptured and in a very bad condition. The posterior tibial artery was completely intact and the decision was to tie the anterior tibial artery and preserve the posterior tibial artery. In attempt to preserve the posterior artery, a frequent assessment during the procedure and postoperatively by doppler ultrasound was performed and revealed an intact circulation of the artery.

**Fig. 2:**
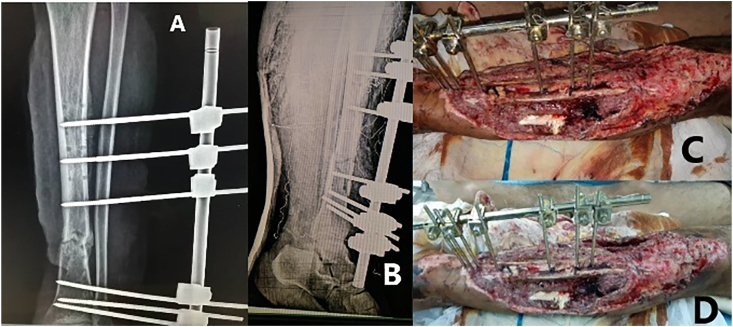
After surgery; anterior (A) and lateral (B) tibial X-ray show external fixator type AO with pins. postoperative clinical photographs showing (C, D) open tibial fracture with soft tissue defect and wasting of the anterior compartment muscles.

In the emergency operation room, the surgery was performed by fifth year resident surgeon under the supervisor view. We did irrigation and debridement and then we placed an anteromedial external fixation system type AO with pins (Fig. 2) and set a posterior slab. Intravenous antibiotics, after discussion with the infectious disease specialists, was consisting of ciprofloxacin, cefazolin and clindamycin, as well as prophylaxis for tetanus.

After the surgery, the patient was transferred to the Orthopedic ward, where he was transfused a red blood cell unit. Intravenous antibiotics were continued and administration of subcutaneous 2.5 mg fondaparinux per day was started. Four days after surgery, antibiotics were switched to meropenem, when he developed fever, elevated c-reactive protein (CRP = 133 mg\dl, up to 6 mg\dl), and cultures of swap and tissue debris came back positive for enterobacter that is sensitive to meropenem.

**Fig. 3:**
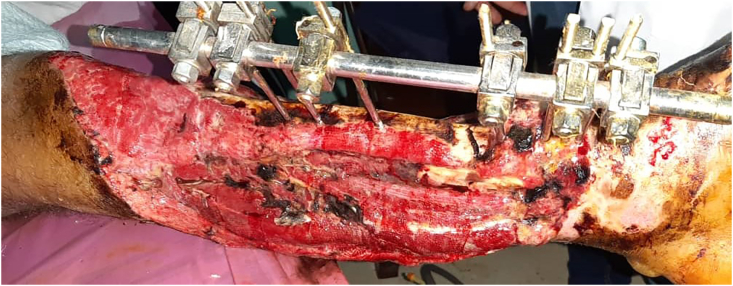
A granular tissue formation three weeks after a daily irrigation, debridement and bandages.

**Fig. 4:**
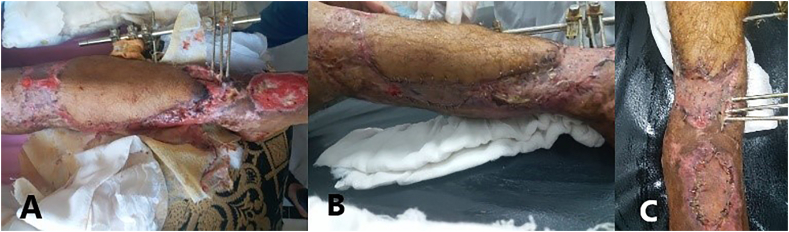
The cross-leg flap and free skin grafts from the opposite limb.

The granular tissue formed after a week of daily irrigation and debridement with povidon bandages followed by two weeks of saline irrigation with ozone cream bandages (Fig. 3). Thereafter, three weeks of orthopedic surgery, the external fixator was translocated to the lateral side and the patient referred to the plastic surgery. The cross-leg flap and free skin grafts from the opposite limb was performed after excluding the infection by CRP, procalcitonin and cultures of the swap and tissue debris. Thereafter, the flap vitality was continuously reassessed and was gradually split in two occasions, three and four weeks after (Fig. 4).

**Fig. 5:**
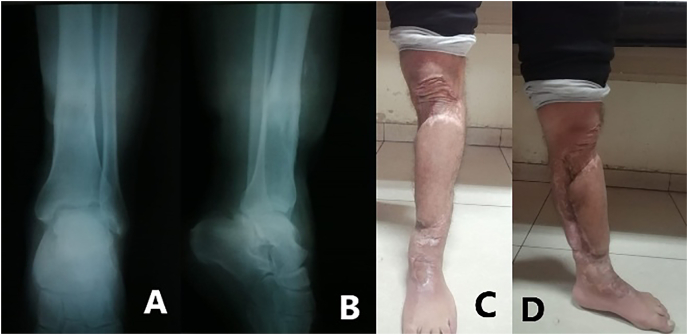
A year after; Anterior (A) and lateral (B) tibial X-ray show a healing tibial fracture. Anterior (C) and lateral (D) tibial clinical photographs show the flap and grafts were healing successfully.

The patient was discharged two months after admission, with a prescription of apixaban (2.5 mg twice a day) and daily povidon bandages. After twelve months of follow-up, the fracture was healed and the external fixator was removed (Fig. 5).

## 3 Discussion

Tibial fractures are seen in approximately 15% of all adult fractures. It is frequently caused by direct or indirect traumas due to slimness of cutaneous and subcutaneous tissues of the anterior tibial shaft. Previous studies have shown that compound fractures are estimated by 23.5% of all tibial shaft fractures [8,9].

Gustilo-Anderson type 3 open fractures remain to be one of the important orthopedic surgery problems because of neurovascular damages, high amputation rate, and vast soft tissue injuries resulting in treatment challenges and complications [3,4]. Historically, open type IIIC tibial fractures have been treated with primary amputation, with studies reporting up to 78% amputation rates [10]. When an open fracture is complicated with vascular injury requiring reconstruction, the management is challenging and the demands are even higher [11].

In this case, since a left open distal tibial fracture (Gustilo type 3C) with a massive loss of muscles, soft tissue, and a disruption of the anterior tibial artery, the emergent choice was an above-knee amputation. However, according to availability of well-trained orthopedic surgeons and a good collaboration with a vascular surgeons, we decided to preserve the left limb and placed an anterior external fixation system type AO with pins. Also, intraoperative and postoperative assessment of the posterior tibial artery by doppler, allowed us to preserve the limb circulation.

For Gustilo type 3B open tibial fractures, reconstruction should ideally be performed within 24 hours using free flap transfer due to the high rate of infections, which is approximately 50%. So, it is important to perform a reconstruction of the bone and soft tissues as soon as possible [4,12]. Injured limbs with failed free flap surgeries are especially more difficult to salvage [13].

In this patient, however, the plastic surgery was delayed for three weeks due to a broad contaminated open fructure and loss of the anterior tibial artery (Gustilo type 3C), it was performed with a cross-leg flap with free skin grafts from the opposite limb without serious complications. Also, immediate antibiotics, that aligned with daily irrigation, debridement, and locally povidon bandages with ozone cream, allowed to salvage the limb. One year of close follow-up, the left limb reclaimed its function and external appearance.

## 4 Conclusion

We report a rare case of a severe damaged left open distal tibial fracture (Gustilo type 3C) with a broad wasting of muscles and soft tissue. The collaboration of the multidisciplinary surgical team led to preserve the limb and support the cross-leg flap in repairing a wide open fracture defect. So, It is reasonable to judge and attempt a limb preservation even with wide open tibial fractures.

## Sources of funding

This research did not receive any specific grant from funding agencies in the public, commercial, or not-for-profit sectors.

## Ethical approval

Written informed consent was obtained from the patient for publication of this case report and accompanying images, in line with local ethical approval requirements and in accordance with the helsinki declaration.

## Consent

Written informed consent was obtained from the patient for publication of this case report and accompanying images. A copy of the written consent is available for review by the Editor-in-Chief of this journal on request.

## Author contribution

Abdullah Alsultan writes the manuscript, literature search, treat and follow up the patient and submitted the article. Mohamad Al Masri writes the manuscript, literature search, treat and follow up the patient. Maher Al-Hajjaj writes and correct the manuscript and search the literature. Muhammad Al Atrash; manuscript correction, literature search and supervisor of the case. Mohammad Alsultan writes and correct the manuscript and search the literature.

## Registration of research studies

1. Name of the registry: N\A
2. Unique Identifying number or registration ID: N\A
3. Hyperlink to your specific registration (must be publicly accessible and will be checked): N\A

## Guarantor

The corresponding author is the guarantor of this manuscript.

## Provenance and peer review

Not commissioned, externally peer-reviewed.

## Declaration of competing interest

The author declares that they have no conflicts of interest regarding this study.

The author declares that it has not been published elsewhere and that it has not been submitted simultaneously for publication elsewhere.
